# Effect of Soluble Adenylyl Cyclase (ADCY10) Inhibitors on the LH-Stimulated cAMP Synthesis in Mltc-1 Leydig Cell Line

**DOI:** 10.3390/ijms22094641

**Published:** 2021-04-28

**Authors:** Thi Mong Diep Nguyen, Laura Filliatreau, Danièle Klett, Nong Van Hai, Nguyen Thuy Duong, Yves Combarnous

**Affiliations:** 1 CNRS, INRAe, Physiologie de la Reproduction et des Comportements, 37380 Nouzilly, France; filliatreau.laura@orange.fr (L.F.); daniele.klett@inrae.fr (D.K.); yves.combarnous@inrae.fr (Y.C.); 2 Faculty of Natural Sciences, Quy Nhon University, Quy Nhon 820000, Vietnam; 3 Institute of Genome Research, Vietnam Academy of Science and Technology, Hanoi 100000, Vietnam; vhnong@igr.ac.vn (N.V.H.); tdnguyen@igr.ac.vn (N.T.D.)

**Keywords:** adenylyl cyclase, cAMP, Leydig cells

## Abstract

In contrast to all transmembrane adenylyl cyclases except ADCY9, the cytosolic soluble adenylyl cyclase (ADCY10) is insensitive to forskolin stimulation and is uniquely modulated by calcium and bicarbonate ions. In the present paper, we focus on ADCY10 localization and a kinetic analysis of intracellular cAMP accumulation in response to human LH in the absence or presence of four different ADCY10 inhibitors (KH7, LRE1, 2-CE and 4-CE) in MTLC-1 cells. ADCY10 was immuno-detected in the cytoplasm of MLTC-1 cells and all four inhibitors were found to inhibit LH-stimulated cAMP accumulation and progesterone level in MLTC-1 and testosterone level primary Leydig cells. Interestingly, similar inhibitions were also evidenced in mouse testicular Leydig cells. In contrast, the tmAC-specific inhibitors ddAdo3′ and ddAdo5′, even at high concentration, exerted weak or no inhibition on cAMP accumulation, suggesting an important role of ADCY10 relative to tmACs in the MLTC-1 response to LH. The strong synergistic effect of HCO_3_^−^ under LH stimulation further supports the involvement of ADCY10 in the response to LH.

## 1. Introduction

Cyclic adenosine 3′,5′-monophosphate (cAMP) is synthesized from ATP through catalysis by adenylyl cyclases (ADCY) and degraded by catabolizing phosphodiesterases (PDEs). In mammals, there are two distinct types of ADCY, nine transmembrane ADCY (tmAC; ADCY1–9) and one soluble (ADCY10) [1]. The tmACs reside at the plasma membrane. They are regulated by heterotrimeric G proteins in response to the stimulation of G protein-coupled receptors (GPCRs) by extracellular hormones and neurotransmitters [2]. In contrast, ADCY10 is found throughout the cytoplasm, and in various organelles, including the nucleus and mitochondria matrix [1,3]. The ADCY10 is insensitive to G proteins and forskolin (a tmAC activator) [4] but is directly activated by calcium and bicarbonate [5,6]. Structurally, ADCY10 and tmACs are monomeric proteins, which catalyze cAMP production through dimerization of the C1 and C2 domains forming their catalytic site [7]. The ADCY10 is soluble in the cytoplasm because it lacks the two hydrophobic domains, each including six membrane-spanning helices anchoring tmACs in the plasma membrane [8]. The nine tmACs (ADCY1-9) and the sole soluble ADCY10 are expressed in various proportions in mammalian cells, so that many cells express both ADCY types. Therefore, to fully understand the regulation of cAMP signaling pathways, it is essential to discriminate the relative contributions of ADCY10 and tmACs.

Using a genetic method, the physiological roles of ADCY10 have been identified through two ADCY10 knockout (KO) mouse strains [9,10,11], and ADCY10-specific siRNA [12,13,14]. It can also be distinguished using pharmacological ADCY10 inhibitors such as catechol derivatives of estrogen (CEs) and KH7 [1,15]. CEs inhibit ADCY10 by binding to a hydrophobic cleft and chelating metal cofactors [16]. CEs have been used to understand the role of ADCY10 in cAMP dependent processes, but they are not that useful to do so because they can also inhibit tmACs [17]. KH7 has been identified in a molecular screen against purified ADCY10 protein [10]. Being more specific for ADCY10 than for tmACs [15], it is the most often used reagent for identifying cAMP signaling functions mediated by ADCY10. The mechanism of action of KH7 is not known yet, but it has been established that it possesses non-specific (off-target) cellular effects [18,19]. LRE1 is the most specific ADCY10 inhibitor, which acts by occupying the binding site of its physiological activator HCO_3_^−^ with no toxicity [20].

MLTC-1 (mouse Leydig tumor cell-1) originated from a spontaneous Leydig cell tumor and were selected in 1982 with the aim of obtaining a Leydig cell model with maintained hormonal response [21]. Leydig cells in the testis are the main source of testosterone in mammalian species. The synthesis and secretion of this steroid is controlled essentially by the luteinizing hormone (LH) produced by the hypophysis. Binding of LH to its G-protein-coupled receptor (LHR) on Leydig cells activates adenylyl cyclases, triggering an increase in cyclic AMP (cAMP) levels from the intracellular ATP source to promote steroidogenesis [22]. Therefore, in this study, we were particularly tested the effects of several ADCY10 and tmAC inhibitors to get more precise information concerning the role of ADCY10 in the Leydig MLTC-1 response to LH.

## 2. Results

### 2.1. Identification and Localization of ADCY10 in MLTC-1 Cell Line

To consider the possible involvement of ADCY10 in the modulation of cyclic AMP response to LH in MLTC-1 cells, we have first checked for its presence by western blot and indirect immunofluorescence, using specific primary antibodies against ADCY10.

**Figure 1:**
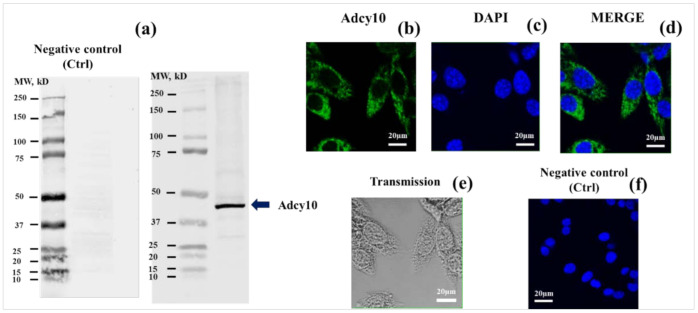
Soluble adenylyl cyclases (ADCY10) immunodetection in MLTC-1 cells. (**a**) Western-blot dentification of ADCY10 in MLTC-1. Cell lysates were resolved by SDS-PAGE, transferred to nitrocellulose membrane and then, probed with an anti-ADCY10 antibody. Loading the first lane of the well with Protein Marker (Precision Plus Protein Dual Color Standards). This marker has three reference bands that allow to monitor electrophoresis. When the gel is stained, 10 bands are visible ranging from 10–250 kDa. We prepared two gels at the same time under the conditions described in the Materials and Methods section. Gel 1 (**a**-right) was then stained with anti-ADCY10 antibody, but gel 2 (**a**-left) was not, in order to check the specificity of the second antibody, anti-rabbit IgG (H + L) (CF770 Conjugate). Membranes scanned using the Odyssey CLx were imaged using Image Studio Software 5x (https://www.licor.com/bio/software (accessed on 1 November 2019)); Indirect immunofluorescence of MLTC-1 cells was carried out with the same antibody. Immunofluorescence staining of ADCY10 in green (**b**) was conducted. Nuclei were stained in blue with DAPI (**c**). Merged images of fluorescence staining are shown in fig (**d**). Transmission images in MLTC-1 cells (**e**). Negative control (Ctrl): primary antibody is missing (**f**). Scale bar: 20 μm. Image analysis was performed using ImageJ software version 1.52a bundled with 64-bit Java 1.8.0_172 (http://rsbweb.nih.gov/ij/ (accessed 1 July 2018)).

Western blotting for ADCY10 in MLTC-1 cell revealed a band with an apparent molecular mass of 48 kDa (Figure 1a). Immunofluorescence data show the presence of ADCY10 throughout the cytoplasm of MLTC-1 cells (in green) (Figure 1b,d). Figure 1e was processed for TEM (Transmission). Negative control was performed by omitting primary antibodies against ADCY10 (Figure 1f).

### 2.2. KH7, LRE1, 2-CE or 4-CE Treatments Reduce the Intracellular cAMP Response to LH in MLTC-1 Cells

We examined ADCY10 activity by assessing the effect of its specific inhibitors on the LH- stimulated cAMP accumulation in cells. In this study, we used MLTC-1 cells transiently expressing a chimeric cyclic AMP-responsive luciferase so that real-time variations of intracellular cyclic AMP concentration could be followed using oxiluciferin luminescence produced from catalyzed luciferin oxidation [23]. The responses to hLH in the different conditions were evaluated using areas under the curves (AUC) of their kinetics over 1 h stimulations. To determine whether KH7, 2-CE, 4-CE, or LRE1 affect the cAMP response to 0.7 nM hLH (2 ng/well), MLTC-1 cells were pre-treated for 1 h in the absence (control, 0 µM) or presence of KH7, 2-CE, 4-CE, or LRE1 before the addition of hLH.

**Figure 2:**
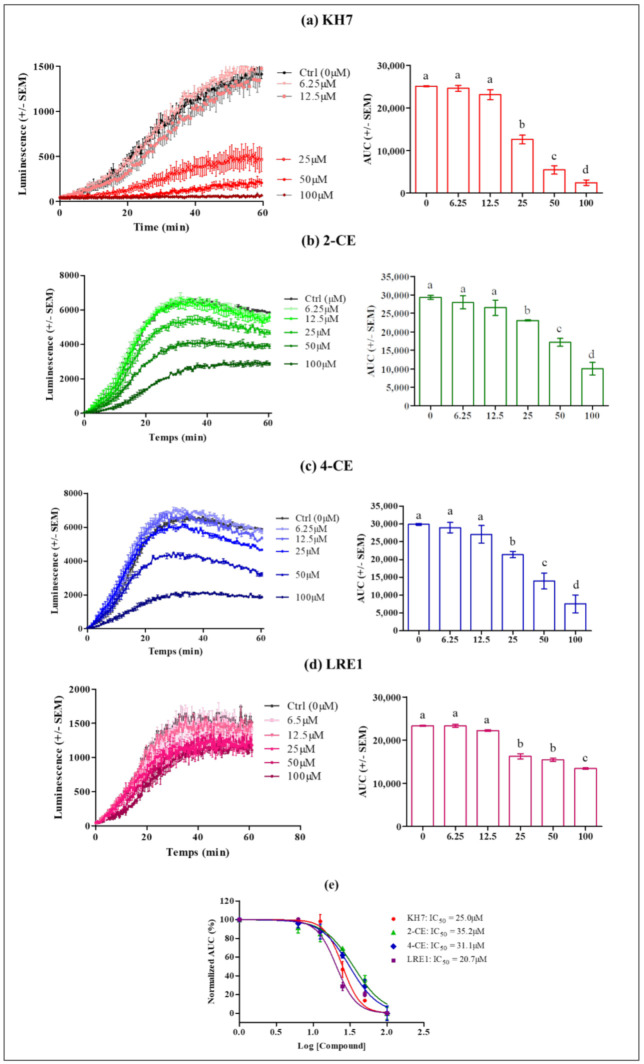
Effect of KH7, 2-CE, 4-CE and LRE1 on the intracellular cAMP response to 0.7 nM hLH in MLTC-1 cells. Cells were treated with the indicated 6.25, 12.5, 50, and 100 µM concentrations of (**a**) KH7, (**b**) 2-CE, (**c**) 4-CE, or (**d**) LRE1. The AUC values are means ± SEM for *n* = 3 independent experiments. *p* value of < 0.05 was considered statistically significant using a one-way ANOVA followed by the Dunnett’s test posttest. Different letters indicate significant differences between control (0 µM) and treatment at *p* < 0.05. (**e**) KH7, 2-CE, 4-CE or LRE1 dose-response curves in MLTC-1 cells. The curves represent three independent experiments combined, with error bars showing mean ± SEM. The *y*-axis represents normalized AUC values (%) for the luminescence signal; The *x*-axis denotes Log IC50.

Figure 2 shows the dose-dependent effects of the four ADCY10 inhibitors upon stimulation by 0.7 nM hLH, of transfected cAMP-dependent luciferase. Clear significant KH7, 2-CE, 4-CE or LRE1 dose-dependent decreases of LH responses are observed as calculated by the areas under the curves (AUC). At 100 µM concentration, KH7 almost completely abolishes the LH-stimulated cAMP accumulation responses (Figure 2a), 2-CE decreases it by about 70% (Figure 2b), 4-CE by about 80% (Figure 2c) and LRE1, by only 36% (Figure 2d). The IC50 for KH7, 2-CE, 4-CE and LRE1 were 25.0, 35.2, 31.1 and 20.7 μM, respectively (Figure 2e).

### 2.3. Effect of KH7, LRE1, 2-CE and 4-CE on MLTC-1 Viability and ATP Concentration

**Figure 3:**
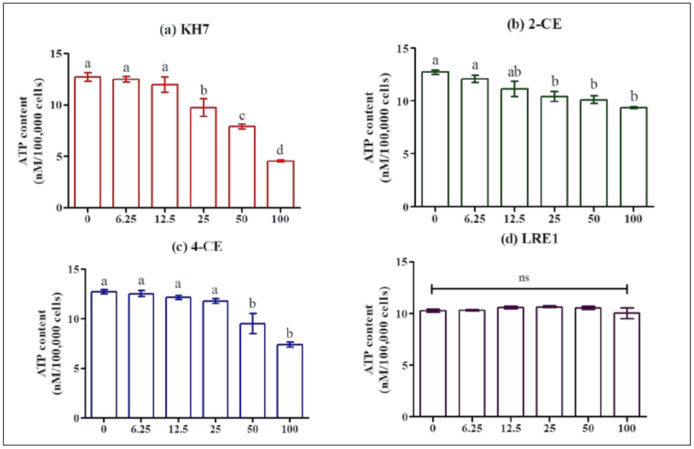
Effect of KH7, 2-CE, 4-CE and LRE1 on the ATP level in MLTC-1 cells. ATP level in MLTC-1 cells was measured after incubation with various concentrations (0, 6.25, 12.5, 50, 100 µM) of KH7 (**a**), 2-CE (**b**), 4-CE (**c**), or LRE1 (**d**) for 1 h. The experiments were repeated 4 times; values are mean ± SEM. Different letters indicate significant differences between control (0 µM) and treatment at *p* < 0.05; ns: not statistically significant.

We checked that the observed effects were not due to decreased cell viability or intracellular ATP availability. We measured ATP levels in MLTC-1 cells after a 1 h incubation in the presence of different concentrations of 2-CE, 4-CE, KH7, or LRE1 (Figure 3). From 25 µM on, KH7 and 2-CE significantly decrease intracellular ATP concentration (Figure 3a,b) whereas 4-CE exhibits the same effect only at 50 and 100 µM (Figure 3c). In contrast to the three other ADCY10 inhibitors, LRE1 up to 100 µM, had no effect on the ATP level (Figure 3d).

**Figure 4:**
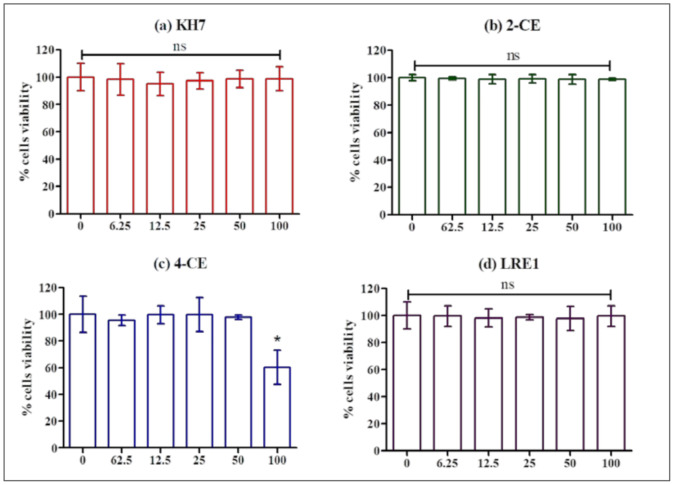
Effect of KH7, 2-CE, 4-CE and LRE1 on the viability of MLTC-1 cells. The viability of MLTC-1 cells was measured after incubation with various concentrations (0, 6.25, 12.5, 50, 100 µM) of KH7 (**a**), 2-CE (**b**), 4-CE (**c**), or LRE1 (**d**) for 1 h. The experiments were repeated 4 times; values are mean ± SEM. Asterisks indicate significant differences between control (0 µM) and treatment at *p* < 0.01; ns: not statistically significant.

After a 1 h incubation in the presence of KH7, 2-CE, 4-CE or LRE1, following a 2 h preincubation with CellTiter-Glo 2.0 Assay substrate, MLTC-1 cells were found to retain full viability with KH7 (Figure 4a), 2-CE (Figure 4b) and LRE1 (Figure 4d), except at the highest 4-CE concentration (Figure 4c).

### 2.4. Fast ADCY10 Activation in MLTC-1 Cells

**Figure 5:**
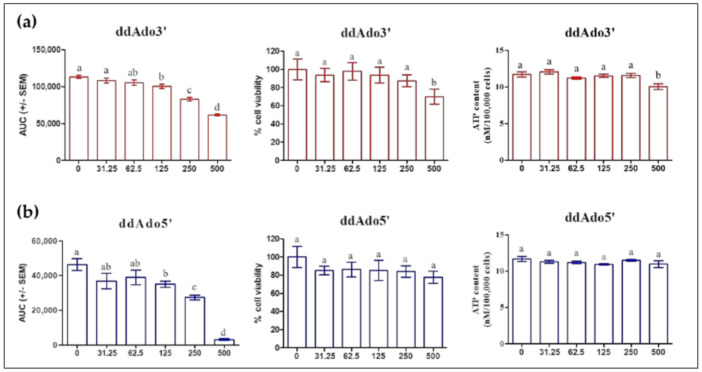
Effect of ddAdo3′ (**a**) and ddAdo5′ (**b**) on the intracellular cAMP response to 0.7 nM hLH, on the cell viability and ATP concentration. Cells were treated with various concentrations (0, 31.25, 62.5, 125, 250, 500 µM) of ddAdo3′ or ddAdo5′ for 1 h. The values are means ± SEM for *n* = 3 independent experiments. *p* value of <0.05 was considered statistically significant using a one-way ANOVA followed by the Dunnett’s test post-test. Different letters indicate significant differences between control (0 µM) and treatment at *p* < 0.05.

In order to distinguish tmAC and ADCY10 stimulation, we tested two cell-permeable selective p-site inhibitors of tmAC: ddAdo3′ and ddAdo5′. Figure 5 shows that ddAdo5’ significantly inhibited cAMP from only 125 µM up to 500 µM without causing cell death in MLTC-1 cells. In contrast, ddAdo3′caused cell death at concentration of 500 µM. Both ddAdo3′ and ddAdo5′ did not affect intracellular ATP concentration.

**Figure 6:**
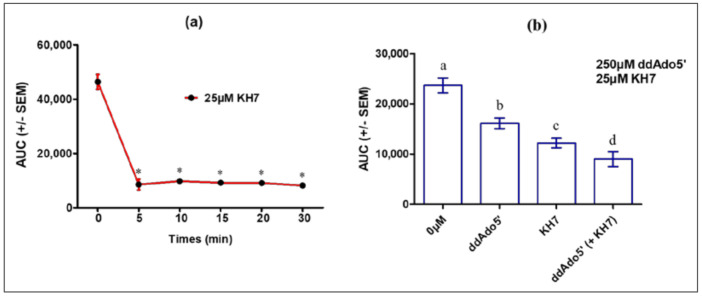
Effects of KH7 and ddAdo5′ on the intracellular cAMP response to 0.7 nM hLH in MLTC-1 cells. (**a**) Kinetics of KH7 action: MLTC-1 was incubated with 25 µM KH7 for 0–30 min before the addition of hLH and luminescence was recorded for 30 min after hLH introduction; (**b**) Effects of separate or concomitant actions of KH7 and ddAdo5′. MLTC-1 was incubated with 250 μM ddAdo5′ for 25 min and then exposed to 25 µM KH7 for 5 min. The values are means ± SEM for *n* = 3 independent experiments. *p* value of < 0.05 was considered statistically significant using a one-way ANOVA followed by the Dunnett’s test post-test. Different letters or asterisk indicate significant differences between control (0 µM) and treatment at *p* < 0.05. ns = not statistically significant.

Thus, to determine the balance between tmAC and ADCY10 roles in cAMP accumulation in MLTC-1 cells under LH stimulation, we tested the effects of KH7 alone or together with ddAdo5′. A short preincubation (5 min) with 25 µM KH7 was sufficient to fully abolish the response to 0.7 nM hLH (Figure 6a). The hLH-stimulated cAMP accumulation in MLTC-1 cells nevertheless was also inhibited by ddAdo5′ but, at a ten-fold higher concentration (250 µM) (Figure 6b).

### 2.5. Bicarbonate (HCO_3_^−^) Potentiates the Intracellular cAMP Response to LH in MLTC-1 Cells

**Figure 7:**
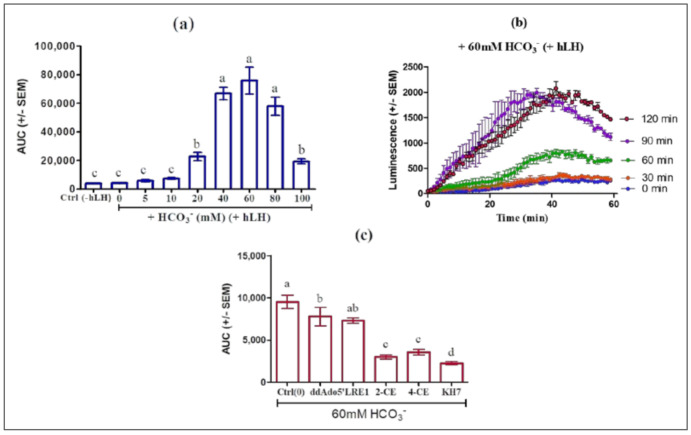
Inhibition of LH/HCO_3_^−^ stimulation of MLTC-1 cells by soluble adenylyl cyclases (ADCY10) inhibitors KH7, 2-CE, 4-CE and LRE1. (**a**) Dose-dependent effect of HCO_3_^−^ on the LH-stimulated intracellular cAMP accumulation in MLTC-1. The cells were preincubated for 60 min in HEPES buffer solution in the presence of different HCO_3_^−^ concentrations before stimulation by 0.7 nM hLH and Control (Ctrl) (absence of HCO_3_^−^ and hLH). (**b**) Kinetics of LH-stimulated intracellular cAMP accumulation after a 0 to 120 min preincubation in HEPES buffer solution in the presence of 60 mM HCO_3_^−^. (**c**) Effects of different ADCY10 inhibitors on the LH-stimulated intracellular cAMP accumulation in MLTC-1. The cells were preincubated in HEPES buffer solution with 60 mM HCO_3_^−^ for 90 min and then exposed to KH7 (50 µM), 2-CE (100 µM), 4-CE (100 µM) or LRE1 (100 µM) or ddAdo5’ (250 µM) for 30 min, before stimulation by 0.7 nM hLH. The values in all experiments are means ± SEM for *n* = 3 independent experiments. *p* value of <0.05 was considered statistically significant using a one-way ANOVA followed by the Dunnett’s test post-test. Different letters indicate significant differences between control (0 µM) and each treatment at *p* < 0.05.

HCO_3_^−^ stimulates ADCY10 by accelerating the renewal of its substrate ATP via an allosteric change leading to closure of the active site, recruitment of catalytic Mg^2+^, and rearrangement of phosphates in bound ATP, as demonstrated in the ADCY10 homologue of cyanobacterial adenylyl cyclase [5,6]. HCO_3_^−^ may prepare the active site for productive ATP binding and support catalysis and PPi release. HCO_3_^−^ stimulates ADCY10 activity by triggering release of the Arg176 thus forming the open cationic state of the catalytic site [24]. In this experiment, we incubated MLTC-1 cells with HCO_3_^−^ at different concentrations and monitored its effect on cAMP response alone or in the presence of 0.7 nM hLH. Our results show that HCO_3_^−^ alone had no stimulatory effect on intracellular cAMP accumulation and when pre-incubated with the cells for 60 min, HCO_3_^−^ at 60 mM maximally increased the cAMP response to hLH (Figure 7a) and that the pre-incubation time for optimal stimulation effect is 90 min (Figure 7b).

In order to ascertain that the observed changes were actually due to ADCY10 activation, we compared the effects of the four ADCY10 inhibitors KH7, 2-CE, 4-CE, or LRE1 and of the tmAC inhibitor ddAdo5’, in the presence of HCO_3_^−^. MLTC-1 cells were pre-treated with 60 mM of HCO_3_^−^ for 60 min, and then treated with KH7 (50 µM), 2-CE (100 µM), 4-CE (100 µM), LRE1 (100 µM), or ddAdo5’ (250 µM) for 30 min. The increases in cAMP response to 0.7 nM hLH by HCO_3_^−^ were significantly attenuated by KH7, 2-CE, 4-CE and LRE1 but not by ddAdo5’ (Figure 7c).

### 2.6. KH7, LRE1, 2-CE and 4-CE Inhibit hLH-Induced Progesterone Secretion in MLTC-1 Cells and Testosterone Secretion in Mouse Testicular Leydig Cells

Because it is difficult to get highly purified mouse testicular cells and to transfect them with the cAMP-dependent luciferase vector, we rather chose to look at the effects of ADCY inhibitors on their testosterone secretion in response to LH. Indeed, it has been established a long time ago that testosterone secretion in Leydig cells is stimulated by intracellular cAMP. To get a proper control, we also studied the effects ADCY10 inhibitors on the progesterone secretion of MLTC in response to LH as this cell line secrete much more progesterone than testosterone [25].

**Figure 8:**
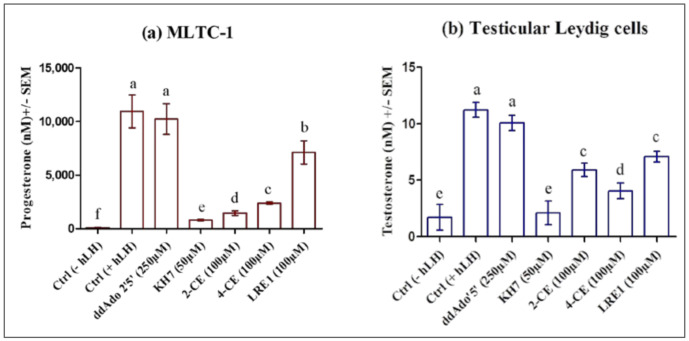
Effect of KH7, 2-CE, 4-CE, LRE1, or ddAdo5′, on 0.7 nM hLH-promoted steroid production in MLTC-1 and primary Leydig cells. (**a**) MLTC-1 cells or (**b**) Testicular Leydig cells were preincubated with or without KH7, 2-CE, 4-CE, LRE1, or ddAdo5′, at the indicated concentrations for 1 h and then the cells were stimulated for 3 hours with 0.7 nM hLH before progesterone (**a**) or testosterone (**b**) productions were measured. Data are means ± SEM of 3 independent experiments performed in duplicate. *p* value of <0.05 was considered statistically significant using a one-way ANOVA followed by the Dunnett’s test post-test. Different letters indicate significant differences between control (0 µM) and each treatment at *p* < 0.05.

All four ADCY10 inhibitors, KH7, LRE1, 2-CE, and 4-CE but not the tmAC inhibitor ddAdo5′, reduced the LH-stimulated progesterone production in MLTC-1 cells (Figure 8a) as well as the testosterone production in testicular Leydig cells (Figure 8b).

## 3. Discussion

The present study demonstrates the presence of ADCY10 in the MLTC-1 cells and that it plays an important role in the cAMP intracellular accumulation under LH stimulation. By immunoblot, we identified one single immunoreactive band, at 48 kDa, that is different from what has been described in mouse liver [26], guinea pig colonic epithelial cells [27], or mouse pancreas [28], with two bands at 48 and 34 kDa. Our results also show a rapid inhibition of cAMP synthesis and progesterone secretion in MLTC-1 cells after preincubation with each of the ADCY10-specific inhibitors KH7, 2-CE, 4-CE, or LRE1.

The ADCY10 inhibitors tested in the present study, namely KH7, 2-CE, 4-CE, and LRE1, provoked a strong inhibition of the LH-stimulated cAMP accumulation in MLTC-1 cells. However, the potencies of the different compounds were different, with KH7 exerting the strongest inhibiting effect and LRE1 the weakest. We also pointed out that the decrease in cAMP accumulation using KH7, 2-CE, or 4-CE, was paralleled by a decrease in ATP concentration, suggesting that at least part of the drop in cAMP accumulation provoked by these products could not be due to direct ADCY10 inhibition. By contrast, LRE1 reduced cAMP accumulation without affecting ATP [20], in full agreement with a direct effect on ADCY10. Previous research has also showed that the two CEs and KH7 inhibit mitochondrial ATP production: CEs by decreasing respiration, and KH7 also by reducing respiration at low concentrations and by perturbing membrane potential at high concentrations [5]. In addition, CEs are known to also inhibit tmACs [15,16,17] by binding to a hydrophobic cleft out of the catalytic site, and also by chelating important metal cofactors [17]. The requirement for metal cofactors as well as the CE binding hydrophobic pocket are present in both mammalian ADCY10 and tmACs. It is therefore not unexpected that CEs inhibit both families of adenylyl cyclase in vitro [17]. In contrast, KH7 does not affect tmAC, but has adverse effects on β-cell metabolism, limiting its usefulness in whole-cell ADCY10 studies [19]. Finally, LRE1 is not only a ADCY10 specific inhibitor with high potency, selectivity, stability, and solubility, but also without cytotoxicity compared to KH7 [20]. Under our experimental conditions, LRE1 showed a lower inhibition potency on LH-stimulated cAMP accumulation than the other three ADCY10 inhibitors, but this can be attributed to the off-target effects by the CEs and KH7, independently from ADCY10 activity. Therefore, the inhibition of LH-stimulated cAMP accumulation by LRE1 strongly supports the involvement of ADCY10 in this pathway downstream of LH receptor activation in MLTC. However, since the inhibition by LRE1 is only partial, it is obvious that another ADCY must also be involved.

The role of ADCY10 is also strongly suggested by supplementing MLTC-1 cells with HCO_3_^−^, a specific ADCY10 activator. Although not effective alone, HCO_3_^−^ increased LH-stimulated cAMP accumulation, and this increase was reduced by either KH7, 4-CE, 2-CE, or LRE1. Among mammalian adenylyl cyclases, the regulation of bicarbonate is unique to ADCY10, and the addition of NaHCO_3_ does not affect tmAC [5]. Our data show that LRE1 decreases the increased cAMP production by HCO_3_^−^ in a weaker way than KH7, 4-CE, or 2-CE. This confirms that the stronger inhibitions by 2-CE, 4-CE and KH7 relative to LRE1 might be due to off-target effects. Then, since the three former inhibitors exhibit stronger inhibition of LH-stimulated progesterone in Leydig cells than the most specific one, LRE1, it can be concluded that LH acts through both pathways, involving one tmAC type and ADCY10. In agreement with this view, additive effects of ddAdo, selective inhibitors of tmAC, have been observed in the inhibition of LH-stimulated cAMP accumulation. Nevertheless, ddAdo only reduces cAMP at 125 µM and greater. Previously, Bitterman et al. recommended to use ddAdo at concentrations ranging from 30 to 50 µM for in vitro assays, where it will fully inhibit tmAC activity while having little or no effect on ADCY10 activity [15]. So we suspect that the decrease in intracellular cAMP caused by 250 µM ddAdo5′ (Figure 6b) might be due to an off-target inhibitory effect on ADCY10.

We also showed that the ADCY10 activity was inhibited by KH7, 2-CE, 4-CE and LRE1 but with 10X higher concentrations than the IC50 values determined with purified recombinant ADCY10 [15] in the presence of the divalent cation Mn^2+^ with 2.5 mM ATP as a substrate [16]. The authors of these previous discoveries also indicated that the IC50 for these inhibitors were 10X higher when tested on cell extracts instead of the purified ADCY10. These latter values are therefore in line with the concentration we used in the present study. In another study, consistent with its effects in whole-cell extracts, KH7 decreased the cAMP production in 4-4 cells with an IC_50_ of 27 ± 6 µM [15]. This result is in agreement with the result of our study (IC_50_ = 25 µM). We therefore suspect that IC50 values might be similar or even higher when tested in intact cells compared to extracts. Further investigation to determine the relative efficiency of the numerous commercially available adenylyl cyclase inhibitors in cells or cell extracts, like that of purified ADCY in acellular context, would be needed to derive more information.

Previous studies have suggested that the intracellular rise of cAMP produced by the bicarbonate-dependent soluble adenylyl cyclase plays a central role in spermatozoa maturation [9]. Esposito et al. showed that targeted disruption of the ADCY10 gene does not affect spermatogenesis but dramatically impairs sperm motility, leading to male sterility [9]. The ADCY10 mutant spermatozoa are characterized by a total loss of forward motility and the inability to fertilize oocytes in vitro [9]. The lack of ADCY10 in the sperm affects the fertilization efficiency. The presence of ADCY10 transcript in primary Leydig cells from adult rat and the role of testosterone in regulation of ADCY10 gene expression was already shown in vivo experiments with adult rats [29]. In the present study, we show for the first time that the ADCY10 is involved in the stimulation of testosterone secretion by LH in mouse Leydig cells as well as that of progesterone in MLTC-1. In addition, the partial effects of ddAdos on LH-stimulated steroid formation in MLTC-1 cells and in testicular Leydig cells supports the hypothesis that ADCY10, and not only ADCY9, must play, a significant role, through cAMP accumulation, in the stimulating effect of LH on steroidogenesis.

## 4. Materials and Methods

### 4.1. Materials

All chemicals were purchased from Sigma-Aldrich (St. Louis, MO, USA) unless otherwise noted. (±)-2-(1*H*-Benzimidazol-2-ylthio)propanoic acid 2-[(5-bromo-2-hydroxy-phenyl)methylene]hydrazide (KH7) was obtained from Tocris Bioscience (Bio-Techne Ltd., Lille, France). The protease inhibitor cocktail was from Roche Diagnostics (Roche Diagnostics, Mannheim, Germany). Tris/glycine buffer (10×), and Precision Plus Protein Dual Color Standards (Cat. No. 1610374) were obtained from Bio-Rad (Bio-Rad, Hercules, CA, USA). Primary antibodies against ADCY10 was purchased from Santa Cruz Biotechnology, Inc. (Santa Cruz, TX, USA). The secondary antibody anti-rabbit IgG (H + L) (CFTM770 conjugated antibodies) was purchased from Biotium (Biotium, Hayward, CA, USA); FluoProbes 448 anti-Rabbit IgG antibodies and FluoProbes 546 anti-Rabbit IgG antibodies were purchased from Interchim (Interchim, Montluçon, France). pGlosensor-22F cyclic AMP plasmid and CellTiter-Blue Cell viability assay (G8080) were from Promega (Promega, Charbonnières-les-Bains, France), XtremeGENE HP DNA transfection reagent was from Roche Diagnostics (Roche Diagnostics France, Meylan, France), the recombinant hLH-C35 hormone was from Serono (Serono, Geneva, Switzerland).

### 4.2. Cell Culture, Plasmids and Transfections

The MLTC-1 (mouse Leydig tumor cell–1) was obtained from the American Tissue and Cell Collection (ATCC, LGC Standards, Molsheim, France). Cell culture, plasmids and transfections were carried out according to the method described in our previous work [23,30]. Cells were cultured at 37 °C and 5% CO_2_ in RPMI-1640 medium (Gibco, Invitrogen, Saint-Herblain, France) supplemented with 10% fetal bovine serum, 50 µg/mL gentamicin, 10 units of penicillin/mL and 10 µg/mL streptomycin. Cells were transfected with pGlosensor-22F cyclic AMP plasmid using X-tremeGENE HP DNA transfection reagent. DNA (100 ng plasmid per well) and X-tremeGENE HP DNA transfection reagent (0.3 µL per well) were mixed together with serum-free RPMI medium and incubated at room temperature for 30 min. This plasmid consists in firefly luciferase sequence fused to that of the protein kinase A cyclic AMP-binding domain in a way that allows control of its enzymatic activity by cyclic AMP. And then the cells were sub-cultured at a density of 100,000 cells/well in 96-well plate (Dutscher, Brumath, France) overnight at 37 °C under 5% CO_2_ before use of the cells in the assays.

### 4.3. cAMP Measurement

Transfection supernatants in 96-well plates were removed and replaced by medium deprived of fetal-calf serum (100 µL/well) containing the luciferase substrate luciferin, containing itself 1 mM IBMX (iso-butyl-methyl-xanthine) to inhibit PDE (nucleotide phosphodiesterase) activity, and thus only measure cAMP biosynthesis, i.e., only AC stimulation [23,30]. The plates were incubated for 1 h before adding KH7, 2-CE, 4-CE, LRE1, ddAdo3′, or ddAdo5′, at various concentrations in a 10 µL volume, and then incubated again for 1 h. Finally, individual stimulating hormone (hLH) was added in a 10 µL volume in triplicate wells to reach 0.7 nM hLH [30]. Cyclic AMP was measured using a Polarstar Optima (BMG Labtech Sarl, Champigny sur Marne, France).

### 4.4. Cell Viability Assessment

Cell viability was determined by CellTiter-Blue Reagent (Promega, Madison, WI, USA) using Spectra Gemini spectrofluorimeter (Molecular Devices, Sunnyvale, CA, USA) at an excitation wavelength of 560 nm and an emission wavelength of 590 nm according to the method described in our previous work [30]. MLTC-1 cells were cultured in 96-well plates with 100,000 cells/well at 37 °C under 5% CO_2_ during two days. Then, the medium was removed and replaced with serum-free medium in the absence (control) or in the presence of KH7, 2-CE, 4-CE, LRE1, ddAdo3′, or ddAdo5′. Afterwards, the samples were incubated for 1 h at 37 °C before adding 20 µL of Reagent to each well and incubating it for 2 more hours at 37 °C. The fluorescent signal from the CellTiter-Blue Reagent is proportional to the number of viable cells.

### 4.5. Adenosine Triphosphate (ATP) Concentration Measurement

ATP concentration in cells was measured by the CellTiter-Glo 2.0 Assay (Promega, Madison, WI, USA) according to the method described in our previous work [30]. Standard was prepared by ATP standard (Promega) using serial dilutions to obtain concentrations of 10^−10^, 10^−11^ and 10^−12^ M. Before ATP concentration measurement, the assay buffer and substrate were equilibrated to room temperature for 2 h, and the buffer was mixed together with the substrate. Cells in 96-well plates were incubated for 1 h without (control) or with KH7, 2-CE, 4-CE, LRE1, ddAdo3′, or ddAdo5′ before adding to 50 μL luciferin/luciferase reagent/well. The plate was mixed for 2 min and incubation was continued for 10 min at room temperature. The luminescence at integration time 1000 (ms) was read using an Ascent Luminoskan Luminometer (Thermo, Villebon-sur-Yvette, France) with PBS as a blank for each experiment.

### 4.6. Western-Blotting Analysis

Lysates of MLTC-1 cells were centrifuged at 13,000× *g* for 30 min at 4 °C and the protein concentration in each supernatant was determined by a colorimetric assay (Bio-Rad DC Protein Assay; Bio-Rad). The proteins were separated by 10% SDS-PAGE (SDS Polyacrylamide Gel Electrophoresis) and then transferred onto nitrocellulose membrane (Whatman Protran, Dassel, Germany) [30]. The membrane was blocked by blocking buffer and incubated with anti-ADCY10 (48 kDa), diluted in 5% BSA in TBS-Tween 0.1% (final dilution 1:1000) as primary antibodies overnight at 4 °C. Finally, the membranes were further incubated for 1 h in anti-rabbit IgG (H + L) (CF770 Conjugate) (final dilution 1:2000). Membranes were then scanned on the LI-COR Bioscience Odyssey CLx imaging system (Lincoln, NE, USA).

### 4.7. Immunocytochemistry

MLTC-1 cells at 10^6^ cells/mL in 96-well plates (50 µL/well) were washed 3 times with 1× phosphate buffered saline (PBS), fixed in ice-cold paraformaldehyde (4%) for 4 min. Cells were then washed in PBS (3 × 3 min) and permeabilized with 0.5% Triton X-100 (Sigma-Aldrich) in PBS for 10 min. Non-specific binding was blocked with PBS supplemented with 10% donkey serum for 30 min at room temperature. Cells were then incubated overnight at 4 °C with anti-ADCY10 diluted 1:100 in PBS-1% donkey serum. After 3 rinses with PBS, cells were incubated with Alexa Fluor 488 goat anti-rabbit IgG antibody (1:500 in PBS) for 1 h at room temperature in the dark, rinsed (3 × 3 min) with PBS and incubated with 4′,6′-diamidino-2-phenylindole (DAPI, 0.05 µg/mL, Sigma–Aldrich) for 10 min [30]. The presence of ADCY10 in cells was examined by confocal microscopy using a LSM 700 confocal microscope (Zeiss, Jena, Germany). Negative controls were performed by omitting primary antibodies. Image analysis was performed using ImageJ software v. 1.52a (http://rsbweb.nih.gov/ij/ (accessed on 1 July 2018)).

### 4.8. Progesterone Production Measurement.

MLTC-1 cells were cultured in 96-well plates at 100,000 cells/ well for 3 days and then re-suspended in serum-free RPMI for 1 h and stimulated with 0.7 nM hLH in the absence or presence of KH7, 2-CE, 4-CE, LRE1, or ddAdo5′, or only with serum-free RPMI (vehicle) for 4 h. The supernatant was then stored at −20 °C until ELISAs were performed.

Progesterone levels were quantified by competitive ELISA assay using the same method as in our previous work [30]. A 96-wells plate was coated overnight at 4 °C with a goat anti-mouse IgG antibody at 10 ng/well (UP462140, Interchim, Montluçon, France). The plate was then washed 3 times × 3 min in PBS 1× containing 0.1% Tween 20. Non-specific sites were saturated in PBS-Tween 20 supplemented with 0.2% BSA (200 µL/well) for 1 h at room temperature. Standard progesterone (Q2600, Steraloids, Newport, RI, USA) diluted by PBS-Tween 20-BSA (25 µL/well of 0, 0.78, 1.56, 3.12, 6.25, 12.5, 25, 100 (ng/mL)) or cells supernatants (25 µL/well of 1:300 dilution) were then distributed on this plate. Progesterone-11-Hemisuccinate-HRP (Interchim) was then added, together with 36 ng/well of mouse anti-P4 antibody (AbD Serotec, Biogenesis, Interchim). The plate was incubated for 4 h at room temperature, washed 3 times and 100 µL/well of TMB substrate (Interchim) was added. The mixture was incubated for 20 min at room temperature in the dark. The reaction was stopped with 2 N H_2_SO_4_ and absorbance was measured at 450 nm using Sunrise Absorbance Reader (Tecan, Männedorf, Switzerland).

### 4.9. Primary Culture of Mouse Testis Cells

The testes were collected from 8 to 10 week-old male RjOrl: SWISS (Janvier Labs, Le Genest-Saint-Isle, France) mice according to the method described in in our previous work [30]. It was done immediately after sacrifice by CO_2_-controlled asphyxia using a TEM device (AETEM1). This procedure has been approved by the Ethics Committee of the Val de Loire Center (CNRS, INRAe, Universities of Tours and Orléans (France)). After removing albuginea, testicular tissue was placed in RPMI-1640 medium. Then it was cut into small pieces using a scalpel in a Petri dish containing the same medium. Subsequently, the fragments were transferred in RPMI-1640 medium (4 mL/testicle) and gently stirred for 10 min at room temperature using a small magnetic strip. The scattered cells were separated from the fragments using a Pasteur pipette. All experiments were carried out in accordance with relevent guidelines and regulations.

### 4.10. Testosterone Production Measurement

Testosterone levels were measured using an HTRF-based assay Kit (CisBio Bioassays, Codolet, France) using the same method as in our previous work [30]. Primary Leydig cells in serum-free RPMI were cultured in 96-well plates at 120,000 cells/well and then incubated for 1 h with or without KH7, 2-CE, 4-CE, LRE1, or ddAdo5′. They were then stimulated with hLH (0.7 nM), or only with serum-free RPMI (control) for 3 h at 35 °C. Afterwards, 10 µL of culture supernatant were transferred to a 384-well white microplate and 5 µL of testosterone-XL665 + 5 µL of anti-testosterone-Ey3 + cryptate antibody were added. The 384-well microplate was then incubated at room temperature in the dark for 1 h, excited with a Mithras LB 943 plate reader (Berthold Technologies GmbH & Co. Wildbad, Germany) at 320 nm, and finally fluorescence was measured at 620 nm and 665 nm.

### 4.11. Area under Curve (AUC) Calculations and Statistical Analysis

The GraphPad 5 package (GraphPad Software, San Diego, CA, USA) was used for the Area Under Curve (AUC) determinations of individual kinetics. Mean and SEM values for each triplicate AUCs were determined. Statistical analysis was done using one-way ANOVA followed by the Dunnett’s test post-test. For all statistical analysis, *p* < 0.05 was considered significant.

## 5. Conclusions

Taking into account that there is no cAMP accumulation in MLTC-1 cells under stimulation by 10 µM forskolin, only the ADCY9 (one of the tmAC) and ADCY10 (the only soluble AC) can potentially be involved. Neither ddAdo nor LRE1 (respectively specific for tmAC and solAC) fully inhibit the LH-stimulated cAMP accumulation. It can therefore be suggested that both ADCY9 and ADCY10 and only these two ADCY are involved in the pathway downstream of LH receptor activation in MLTC1. Our preliminary data of testosterone secretion decrease by ADCY inhibitors suggest that this also holds true in testicular mouse Leydig cells.

## Data Availability

Not applicable.
